# HIV-1 Quasispecies Delineation by Tag Linkage Deep Sequencing

**DOI:** 10.1371/journal.pone.0097505

**Published:** 2014-05-19

**Authors:** Nicholas C. Wu, Justin De La Cruz, Laith Q. Al-Mawsawi, C. Anders Olson, Hangfei Qi, Harding H. Luan, Nguyen Nguyen, Yushen Du, Shuai Le, Ting-Ting Wu, Xinmin Li, Martha J. Lewis, Otto O. Yang, Ren Sun

**Affiliations:** 1 Department of Molecular and Medical Pharmacology, David Geffen School of Medicine, University of California Los Angeles, Los Angeles, California, United States of America; 2 Molecular Biology Institute, University of California Los Angeles, Los Angeles, California, United States of America; 3 Department of Microbiology, Immunology, and Molecular Genetics, David Geffen School of Medicine, University of California Los Angeles, Los Angeles, California, United States of America; 4 Department of Microbiology, Third Military Medical University, Chongqing, China; 5 Department of Pathology and Laboratory Medicine, David Geffen School of Medicine, University of California Los Angeles, Los Angeles, California, United States of America; 6 Division of Infectious Diseases, Department of Medicine, David Geffen School of Medicine, University of California Los Angeles, Los Angeles, California, United States of America; 7 AIDS Institute, University of California Los Angeles, Los Angeles, California, United States of America; 8 AIDS Healthcare Foundation, Los Angeles, California, United States of America; Tel Aviv University, Israel, Israel

## Abstract

Trade-offs between throughput, read length, and error rates in high-throughput sequencing limit certain applications such as monitoring viral quasispecies. Here, we describe a molecular-based tag linkage method that allows assemblage of short sequence reads into long DNA fragments. It enables haplotype phasing with high accuracy and sensitivity to interrogate individual viral sequences in a quasispecies. This approach is demonstrated to deduce ∼2000 unique 1.3 kb viral sequences from HIV-1 quasispecies *in vivo* and after passaging *ex vivo* with a detection limit of ∼0.005% to ∼0.001%. Reproducibility of the method is validated quantitatively and qualitatively by a technical replicate. This approach can improve monitoring of the genetic architecture and evolution dynamics in any quasispecies population.

## Introduction

Many viruses have such high replication and mutation rates that they exist as a quasispecies *in vivo* [1]. A viral quasispecies population contains a variety of genotypic variants that are related by similar mutations and exist in varying abundance depending on their relative fitness within the host environment. In this report, we refer to viral quasispecies as the whole population of genotypic variants, whereas viral sequence is defined as the individual viral variant within quasispecies population. Viral sequence variation in the quasispecies population can be rapidly generated by point mutation and/or recombination [1], [2]. Mutation rates can be as high as in the order of one per replication cycle, in which the progeny virus is unlikely to be identical to its parental template. This diverse array of viral sequences permits robust adaptation and evolution.

Often, genotypes with a particular set of mutations gain a significant fitness advantage through synergistic phenotypic effect among multiple mutations, which is also known as epistasis. Epistasis has an important role in host adaptation and may drive evolution towards drug resistance and immune evasion [3]–[7]. In many cases, virus drug resistance requires two or more mutations in concert, especially when multiple drugs are applied simultaneously [7]–[9]. Therefore, monitoring individual viral haplotypes in the quasispecies populations within patients is important to estimate the risk of viral rebound and further provide customized treatment [10]. Characterizing the population structure of viral quasispecies in the host also helps to understand the evolutionary landscape and *cis*-interactions among genetic elements.

Clonal sequencing has been frequently employed to examine the genetic makeup of individual viruses within a quasispecies population. However, clonal sequencing has a low throughput and a high sequencing cost per nucleotide. It limits the number of viral sequences, hence haplotype variants, being genetically interrogated. On the other hand, next generation sequencing (NGS) technology provides enough throughput and sensitivity to detect very rare viral mutations. Nevertheless, the short read lengths of NGS pose a challenge in reconstruction of individual viral sequences within a viral quasispecies. First of all, it is often difficult to distinguish rare mutations that exist in the quasispecies population with sequencing errors from NGS. Secondly, haplotype phasing is extremely challenging when mutations are sporadic and are separated by long, highly conserved or even completely identical regions. These technical challenges make it extremely difficult to reconstruct viral quasispecies from NGS data.

Existing methods in reconstructing viral quasispecies from NGS platforms rely heavily on computational tools, including the development of read graph-based or probabilistic-based algorithms that utilize the information from overlapping reads [11]–[20]. Although they provide an approximation of haplotype information present in a viral quasispecies, the sensitivity and accuracy vary depending on sequencing error rate and quasispecies diversity. As a result, it is critical to develop a viral quasispecies recontruction method with higher sensitivity and accuracy in both mutation calling and haplotype phasing.

In order to genetically define a viral quasispecies population, we developed a novel analytical technique to assemble short Illumina amplicon sequence reads derived from individual viral sequences. In contrast to algorithmic-based methods for quasispecies reconstruction, tag linkage approach is a molecular-based approach. To the best of our knowledge, this is the first experimental approach that specialized in quasispecies reconstruction. The methodology consists of three key steps: 1) Assigning unique tags to individual viral sequences to distinguish each variant within the viral quasispecies, 2) Controlling the complexity of the library during amplification to ensure sufficient coverage for sampled viral sequences, and 3) Using a tag linkage strategy to deduce the full-length templates from non-overlapping amplicons. Here, we provide a proof-of-concept study by utilizing this approach to genetically characterize an HIV-1 quasispecies population under two conditions: an isolated *in vivo* virus population and the virus population derived from the same chronically infected HIV-1 patient passaged *ex vivo* in cell culture. We achieve a detection limit of ∼0.005% to ∼0.001%. The reproducibility is validated with a technical replicate. Overall, this approach enables accurate haplotype phasing with very high sensitivity.

## Results

### Library Preparation for Sequencing

**Figure 1:**
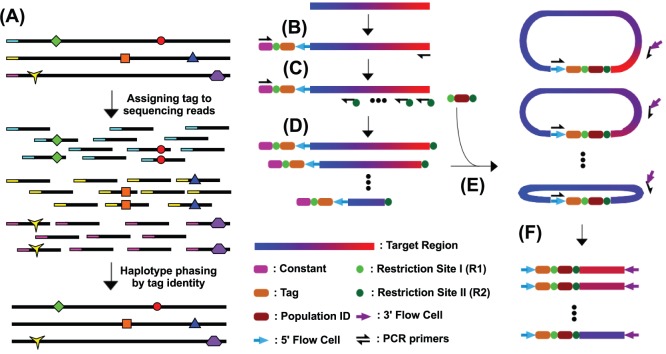
Schematic representation of the experimental design. (A) Individual viral sequences within a quasispecies are assigned with a unique tag. In this example, three viral sequences are present in the quasispecies. Horizontal black lines represent individual viral sequences. Red circle represents mutation from the consensus. Colored tag represents unique tag assigned to individual viral sequences. The same tag is distributed to every sequencing reads originated from the same viral sequence. During quasispecies reconstruction, the tag contains the phasing information to reconstruct individual viral sequences. (B) A cassette consisting of a constant region (Constant), a restriction site (R1), a random oligonucleotide (tag) and the forward Illumina adapter (5′ FlowCell) is added to the 5′ end of the DNA sample. Individual DNA molecules in the resultant pool will each be afforded with a unique tag. (C) The input pool in this PCR step contains a limited number of DNA templates to reduce the complexity of the pool. The resultant PCR amplification generates multiple copies of individually tagged DNA templates. (D) The DNA pool is then divided into a series of PCRs with a second restriction site (R2) added to the 3′ end of the reaction product. (E) Ligation with population ID, which is a short specific DNA sequence serving as a barcode for multiplex sequencing, utilizes the two restriction sites, R1 and R2. (F) Amplicons with similar size are generated from different ligated DNA pools. Reverse Illumina adapter (3′ FlowCell) is added. Different amplicon pools can then be mixed and subjected to Illumina sequencing.

The underlying rationale is to assign a unique tag to individual viral sequences within the quasispecies and to distribute the tag to every sequencing read originated from the same viral sequence (Figure 1A). Individual viral sequences within the quasispecies can be assembled by grouping sequencing reads that share the same tag. As a result, the tag linkage approach described in this study permits reconstruction of individual viral sequences from NGS reads despite the lack of overlap.

The workflow for sequencing library preparation is summarized in Figure 1B–F. Briefly, individual DNA molecules are assigned a unique tag by PCR (Figure 1B). The tag consists of a 13 “N” sequence that allows distinguishing 4^13^ 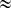 70 million molecules. After tagging individual DNA molecules within the pool, the complexity of the pool is being controlled. Complexity is defined as the number of tagged DNA molecules being processed after the first round of PCR. Thus, the more tagged molecules are being processed, the higher the complexity becomes. If complexity is too high, individual tagged molecules will not be covered repeatedly, leading to a failure in assemble individual DNA molecules (Figure S1A in File S1). On the other hand, if complexity is too low, sequencing capacity will be wasted due to redundant sequencing coverage of individual tagged DNA molecules being processed (Figure S1B in File S1). Nonetheless, for quasispecies determination, it is more detrimental if the complexity is too high versus too low because excessive complexity will abolish the sequence assembly process (Figure S1 in File S1). In general, the relationship between complexity and expected coverage for an individual viral sequence can be calculated with the expected sequencing output:
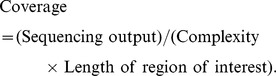



In this formula, sequencing capacity and length of region of interest can be predetermined. Therefore, complexity is estimated solely based on the desired coverage of each tagged DNA molecules. For example, if the region of interest is 1 kb and 1 Gb of sequencing output is expected, then a complexity of 100,000 gives on average 10-fold coverage for individual tagged DNA molecules being processed. With sufficient coverage for an individual viral sequence, we can distinguish sequencing error from true mutation as described previously [21], in addition to haplotype phasing. Therefore, complexity control represents a critical step in our experimental design.

After controlling the complexity, a PCR is performed to generate multiple copies of individually tagged DNA molecules (Figure 1C). The resultant DNA pool is then divided into a series of PCRs to generate products with different lengths (Figure 1D). For every pool, the resultant PCR products contain two different restriction sites on each ends. Next, restriction enzyme digestions generate two sticky ends and remove the constant region for PCR in the earlier step. A self-ligation step follows with the addition of a short insert (Figure 1E). The short insert can serve as a barcode for multiplex sequencing. This ligation step circularizes the DNA, resulting in different sequence regions being proximal to the tag and further allowing linkage formation between any distal region with the tag - another key step in our experimental design. In the final step, a short amplicon (∼200 bp) is recovered for NGS (Figure 1F). Each NGS read, from 5′ to 3′, will cover a tag for short read assembly within a quasispecies sample, a barcode for quasispecies sample identification, and a particular region of interest on the targeted viral sequence. NGS reads sharing the same tag belong to the same DNA molecules. Therefore, haplotypes of individual viral genomes within the quasispecies population can be interrogated. A more detailed schematic representation of the key steps in our approach is shown in Figure S2 in File S1.

### Assembly of Two HIV-1 Viral Quasispecies

Virus derived from a chronically infected HIV-1 patient was analyzed before (*in vivo*) and after (*ex vivo*) cell culture passaging for 10 weeks. *In vivo* virus sample represented the viral quasispecies within the HIV-1 infected patient. Whereas in *ex vivo* passaging, virus from the same patient was passaged serially in primary CD4^+^ T lymphocytes from an HIV-1-uninfected donor and reflected the evolution of the viral quasispecies population in the absence of intra-patient selection pressure. We limited the complexity by processing roughly 300,000 viral sequences to ensure sufficient coverage (∼50-fold) in all regions for any given viral sequence (Figure 1B).
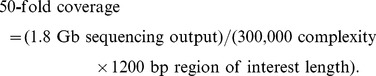



**Figure 2:**
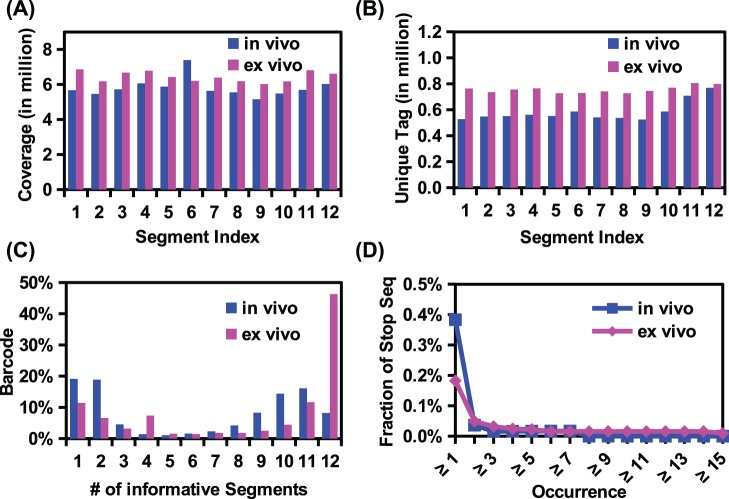
Proof-of-concept study using *ex vivo* passaged HIV-1 quasispecies. (A) Sequence coverage for each of the 12 amplicon segments is plotted. (B) Tag coverage for each of the 12 amplicon segments is plotted. Tag coverage is calculated by the number of unique tags present in a given amplicon segment. (C) The assembling successful rate is assessed by a parameter called the ‘number of informative segments’, which represents the number of amplicon segments a unique tag is present in. For example, in the present study, a tag with 12 informative segments represents a complete assembled contiguous sequence of our HIV-1 DNA target region, while a tag with 11 informative segments indicates that 1 amplicon segment is missing from the assembled contiguous sequence. All tags in the data set are categorized by this parameter. (D) The fraction of sequences containing a stop codon is plotted against different cutoffs for the minimum sequence occurrence.

Twelve non-overlapping amplicons, which cover a 1,295 nucleotide stretch and encompass most of the *gag* and a portion of the *pol* genes of the HIV-1 genome, were prepared. Sequencing was performed using an Illumina HiSeq 2000 machine. Sequencing coverages in different regions were similar (Figure 2A). The numbers of unique tags in different regions were also comparable (Figure 2B). The absence of apparent coverage bias confirmed the quality of sequencing library preparation. For each region, tags with fewer than three occurrences were filtered and removed to adequately apply the error correction algorithm. This filter eliminated 35–57% of tags depending on region. For a complete viral sequence to be assembled, sequences of all 12 amplicon regions sharing the same tag had to be available (Figure S1 in File S1). We successfully assembled 54,583 viral sequences in the *in vivo* viral quasispecies and 228,936 viral sequences in the *ex vivo* quasispecies, thus validating the complexity control procedure (Figure S1 in File S1). However, about ∼30–40% of the tags were present in only one or two regions, which we attributed to PCR or sequencing errors at the tag region (Figure 2C).

To further evaluate the data quality, the appearance of stop codons in *gag* was examined. Given that viable virus requires translation of a full length Gag polyprotein, stop codons would likely represent PCR errors. While ∼0.4% (*in vivo*) and ∼0.2% (*ex vivo*) of the assembled sequences contained a stop codon, this number dropped dramatically (<0.05%) after we filtered-out the sequences with just one occurrence (Figure 2D). Further increasing the cutoff stringency, however, did not significantly suppress the stop codon occurrence frequency. These rare viral sequences were likely to be non-functional virus within the viral quasispecies population generated by hypermutation [22]–[24]. 47,083 assembled viral sequences from the *in vivo* viral quasispecies and 223,966 assembled viral sequences from the *ex vivo* viral quasispecies passed this quality filter, yielding 2,672 and 1,983 unique viral sequences, respectively. The number of unique viral sequences we successfully assembled represented a > 20 fold increased as compared to that of the previously reported algorithm-based quasispecies assembly method [11]–[17]. Additionally, the detection limits of rare viral sequences in this study (∼0.005% and ∼0.001% for the *in vivo* and *ex vivo* viral quasispecies, respectively) also significantly exceeded that reported for the algorithm-based technique, which was reported to be ∼0.1% to ∼1% [15]–[18].

### Comparison with Algorithmic-based Approach

To the best of our knowledge, the existing quasispecies reconstruction approaches are algorithmic-based inference methods. In contrast, tag linkage approach is a molecular-based, direct interrogation method. It is devoid of any inference error that is intrinsic to algorithmic-based approach. Consequently, it enables a much higher accuracy in quasispecies reconstruction than conventional algorithmic-based approach. We compared the performance of our tag linkage method with two algorithmic-based approaches: 1) the state-of-the-art ShoRAH tool [13], and 2) a recently published approach, QuasiRecomb [12], which takes natural recombination event into account. To implement the algorithmic-based approaches, single-read DNA sequencing library of the *in vivo* quasispecies sample was prepared by standard DNA fragmentation. We also employed the tagging strategy here to distinguish true mutations from sequencing error as previously described (see materials and methods) [21]. As a result, quasispecies reconstructions by ShoRAH and QuasiRecomb were minimally confounded by sequencing error. To provide a reference for comparison, we conducted traditional clonal sequencing for the *in vivo* quasispecies population. In this experiment, a 1106 bp region in the *gag* gene was considered. A total of 20 randomly selected clones were sequenced, which represented 14 different haplotypes.

**Figure 3:**
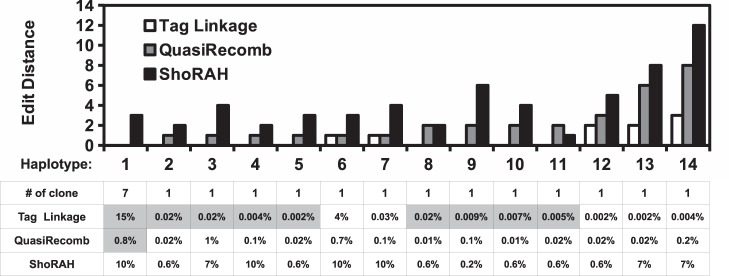
Comparison of performance in quasispecies reconstruction with ShoRAH and QuasiRecomb. A total of 20 randomly selected clones from the *in vivo* quasispecies population were sequenced using traditional clonal sequencing. They represented 14 different haplotypes. Their edit distances with the corresponding closest reconstructed viral sequences are shown. Edit distance represents the minimum number of substitutions required to change one nucleotide sequence into the other. The estimated fractions of closest reconstructed viral sequences for those 14 haplotypes are displayed in the bottom. The estimated haplotype frequency is displayed in a gray background if there is a complete match (edit distance = 0).

ShoRAH reconstructed 252 viral sequences from the *in vivo* quasispecies sample. However, none of the 14 haplotypes were being reconstructed (Figure 3). For those 14 haplotypes, the respective closest viral sequence deduced by ShoRAH had an edit distance ranging from 1 to 12. QuasiRecomb, on the other hand, reconstructed 1343 viral sequence and was able to identify 1 out of 14 haplotypes from clonal sequencing. This haplotype had an estimated occurrence frequency of 0.8% from QuasiRecomb while it accounted for 7 out of 20 clones in clonal sequencing. It implied that haplotype frequency estimation by QuasiRecomb was inaccurate and that a significant amount of reconstructed haplotype by QuasiRecomb was false positive. QuasiRecomb can also be run in a conservative mode, in which only major haplotypes were reconstructed. Under this running mode, only 6 haplotypes were reconstructed and none of them overlapped with the 14 haplotypes being clonal sequenced.

In contrast, 9 out of 14 haplotypes from clonal sequencing were included in the quasispecies reconstructed by our tag linkage approach (Figure 3). The most abundant haplotype from clonal sequencing matched the most abundant reconstructed haplotype from tag linkage approach in this region of interest. The other 8 identified haplotypes were estimated to have an occurrence frequency from 0.002% to 0.02%. It highlighted the sensitivity and accuracy of our tag linkage approach in reconstructing rare haplotypes. The missing five haplotypes were 1–3 edit distances away from their respective closest viral sequence in the quasispecies reconstructed by our tag linkage approach. Overall, tag linkage approach achieved a significant improvement over algorithmic-based approaches in quasispecies reconstruction, both qualitatively and quantitatively.

### Diversity Comparison between *in vivo* and *ex vivo* HIV-1 Quasispecies

**Figure 4:**
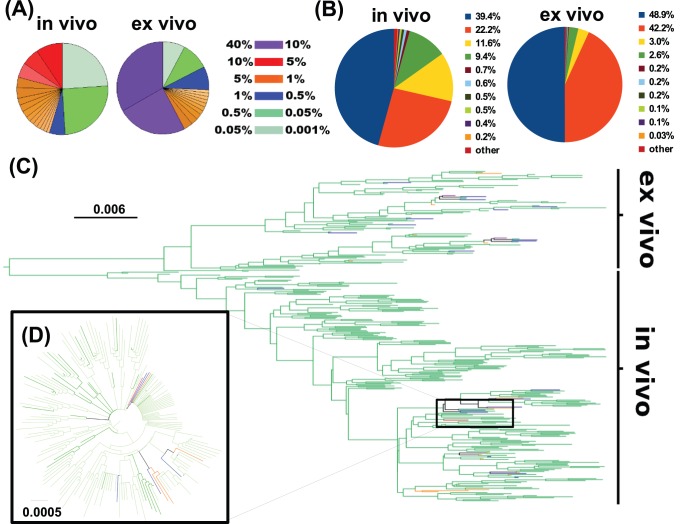
Diversity of *in vivo* and *ex vivo* HIV-1 quasispecies. (A) The diversities for both the *in vivo* and *ex vivo* viral quasispecies at the nucleotide level are reflected by the pie chart. The fractions of viral quasispecies for the 10 highest frequency occurring viral sequences are shown. Each color code indicates a range of occurrence frequency as indicated. (B) The diversities for both *in vivo* and *ex vivo* viral quasispecies content on the protein level (aa. 139 to 507 on *gag* protein) are reflected by the pie chart. The fractions of viral quasispecies for the 10 highest frequency occurring viral sequences are shown. (C) A neighbor-joining phylogenetic tree depicting viral nucleotide sequences with occurrence frequencies above 0.05%. The occurrence frequency for each individual node is color coded as described in Figure 4A. (D) A small segment in the phylogenetic tree from Figure 4C is selected. This segment is replotted along with viral sequences with occurrence frequency form 0.001% to 0.05%, which are not included in Figure 4C.

We next examined the sequence diversity in both *in vivo* and *ex vivo* quasispecies populations. The most frequent viral sequence represented 8.1% of the *in vivo* viral quasispecies, whereas the most dominant viral sequence represented 32.5% of the *ex vivo* viral quasispecies (Figure 4A). The two most dominant viral sequences in the *ex vivo* sample comprised more than half of the total viral quasispecies while the *in vivo* viral quasispecies was much more diverse. At the amino acid level, 80% of the *in vivo* viral quasispecies were represented by four protein sequences, with a total of 42 unique protein sequences in the population (Figure 4B). In contrast, while only two protein sequences represented 80% of the *ex vivo* viral quasispecies, there were 201 unique protein sequences. Table S1 in File S1 provides a summary of this data. A phylogenetic tree analysis demonstrated the effect of differential selection pressures on viral quasispecies evolution from *in vivo* to *ex vivo*, in which two distinct sub-population clusters could be observed (Figure 4C and D).

### Recombination Pattern of HIV-1 Quasispecies

**Figure 5:**
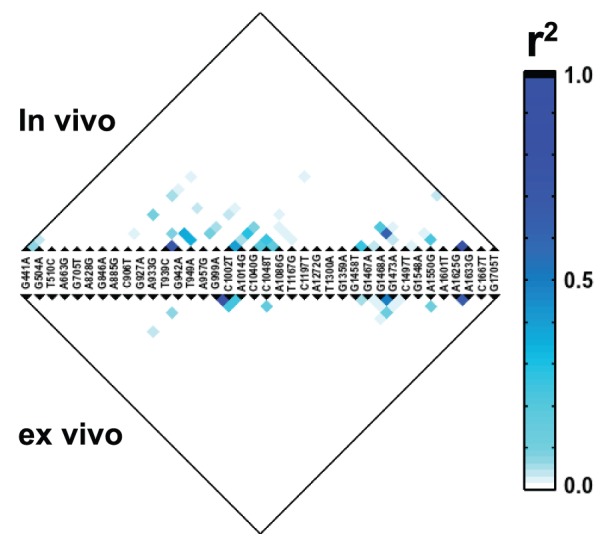
Linkage disequilibrium for *in vivo* and *ex vivo* HIV-1 quasispecies. The linkage disequilibrium was measured using r^2^ and computed between SNPs that had an occurrence frequency above 0.1% in either the *in vivo* and *ex vivo* viral quasispecies. The stronger the association between two SNPs, the larger value the r^2^ is.

HIV-1, as a diploid retrovirus, is capable of generating recombinant proviral transcript via a template switching event during the reverse transcription step in the viral replication. It facilitates further diversification for adaptation [2]. The depth and comprehensiveness of our data permit an investigation of this viral recombination, as a linkage disequilibrium pattern. Here, we employed the r^2^ correlation to measure linkage disequilibrium. r^2^ was computed between 38 SNPs that had an occurrence frequency above 0.1% in either the *in vivo* or *ex vivo* viral quasispecies (Figure 5). Several strong correlations (r^2^ > 0.5) were observed in both the *in vivo* and *ex vivo* viral quasispecies. Nonetheless, the linkage disequilibrium was more pervasive and spanned a larger region in the *in vivo* viral quasispecies than in the *ex vivo* viral quasispecies. From the *in vivo* viral quasispecies, we observed two linkage disequilibrium blocks, a ∼200 nucleotide block from position 900 to 1100 and another from nucleotide position 1400 to 1600. The presence of two closely spaced recombination nucleotide blocks suggests that there is a recombination hotspot between position 1100 to 1400, which is located at the p24 region of the gag gene. Another possibility is that certain haplotypes provided a fitness advantage and were positively selected. Further characterization would be needed to dissect the underlying mechanism.

### Reproducibility from a Technical Replicate

**Figure 6:**
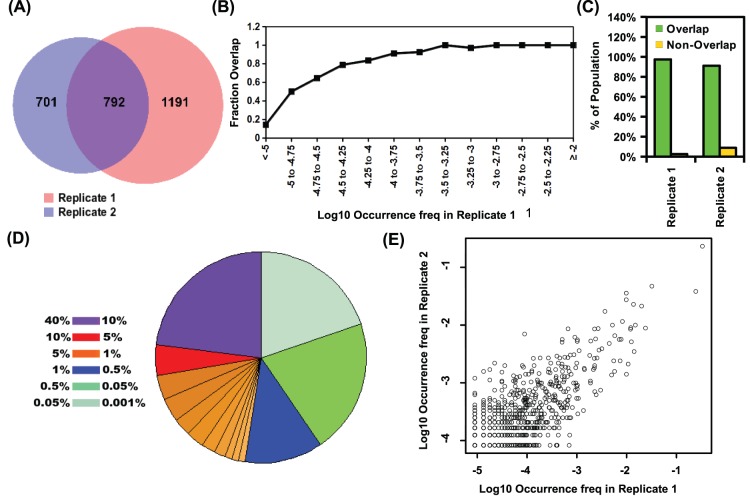
Technical Replicate for *ex vivo* viral quasispecies assembly. (A) The reproducibility was assessed by Venn diagrams of the unique viral sequences in both replicates of the *ex vivo* viral quasispecies reassembly. (B) Unique viral sequences were binned into a bin size of 0.25 at log10 scale. The overlapping faction that was covered by replicate 2 were plotted against different bins. (C) The fractions of viral quasispecies population content in replicate 1 that was covered by replicate 2 (Replicate 1) and in replicate 2 that was covered by replicate 1 (Replicate 2) are plotted as a bar chart. (D) The diversity for replicate 2 at the nucleotide level is reflected by the pie chart. The fractions of viral quasispecies for the 10 highest frequency occurring viral sequences are shown. Each color code indicates a range of occurrence frequency as indicated. (E) The occurrence frequency of individual viral sequences was compared between replicate 1 and replicate 2 at log_10_ scale. There exists a Pearson correlation of 0.87 at normal scale between two replicates.

To assess the reproducibility, a technical replicate was performed for the *ex vivo* viral quasispecies population (Figure 1C–E). The technical replicate was repeated for all steps beginning at the stage of generating amplicons of varying length (Figure 1C) - a key step of our approach. Majority of the viral sequences in the replicate (replicate 2) overlapped with the original data set (replicate 1) (Figure 6A). However, a significant fraction of viral sequences was covered by only one of the replicates, but those represented a small fraction, ∼3% to 9%, of the viral quasispecies (Figure 6B and C). Viral sequences that were observed in only one of the two replicates typically had an occurrence frequency < 0.01% (Figure 6B). It suggests that the difference between replicates was due to sampling limit, where viral sequences with a low occurrence were more likely to be unsampled by one of the replicates. Replicate 2 covered 97% of the viral quasispecies in the first replicate, whereas replicate 1 covered 91% of viral quasispecies in the second replicate (Figure 6C). The genetic composition of the viral quasispecies reconstructed from replicate 2 was comparable to that of replicate 1 (Figure 4A and 6D). Occurrence frequency for individual viral sequences exhibited a correlation of 0.87 (Pearson correlation at normal scale) between replicates (Figure 6E). These results provided further validation of the tag linkage technique in both qualitative and quantitative manners.

## Discussion

With the advancement of sequencing technology, NGS continues to increase read length and throughput. Nonetheless, the trade-off between read length and throughput still exists [25]. Sequencing platforms with long reads such as Pacific Bio and 454 pyrosequencing have a relatively low throughput. NGS machines with higher throughput such as Illumina and SOLiD do not afford long reads. Despite currently having the highest throughput, the short read length of Illumina creates a challenge in assembling reads into continuous long sequences.

This study describes an amplicon-based tag linkage approach to characterize viral quasispecies population structures and provides a proof-of-concept example showing a very high detection sensitivity. Unlike algorithm-based approaches, the accuracy of our amplicon-based molecular tag approach is independent of viral quasispecies population diversity. In addition, it incorporates an error correction step to identify NGS platform errors, resulting in a dramatic increase in the sensitivity to detect rare haplotypes [21]. Algorithm-based approach for viral quasispecies reconstruction can usually handle 10 to 100 viral sequences at various statistical confidence. In contrast, our tag linkage approach can reconstruct close to 1000 sequences with high confidence as indicated by our replicates. It achieves a significant improvement in accuracy and sensitivity from the algorithm-base approach [11]–[20].

The major limitation in our approach is the length of deduced sequence, which is restricted by the upper limit of PCR (typically 10 kilobases). Another potential pitfall is PCR recombination. In our protocol, we tried to minimize this artifact by using a high processivity and fidelity DNA polymerase for PCR [26]. In addition, a long PCR extension time was used to ensure extension completion of the amplicon to minimize PCR recombination [27]. Our technical replicate control shows that a majority of the viral quasispecies population content (>90%) are captured in both repetitions, including rare variants, indicating that any artifact by PCR recombination is minimal. Additionally, the high correlation of occurrence frequency for individual viral sequences between each replicate confirms reproducibility. Overall, our control experiments and concurrent analysis validate the amplicon-based tag linkage approach as a highly sensitive methodology for viral quasispecies assembly.

By reconstructing individual sequences within the viral quasispecies, we are able to detect linkage disequilibrium throughout the region of interest. Genome recombination is a frequent process occurring intra-patient for diversification and adaptation [28]–. Recombinant generation is a non-random process as recombination coldspots and hotspots have been reported in HIV-1 [33]–[36]. In this study, we observed a more pervasive linkage disequilibrium in the *in vivo* viral quasispecies compared to that of the *ex vivo*, suggesting that there may be genetic interactions within the linkage disequilibrium block that are important for chronic infection. Alternatively, this observation may also be attributed to a higher recombination frequency during *ex vivo* passaging due to an increase in co-infection occurrence. We demonstrate the power of our tag linkage approach in capturing linkage disequilibrium in a viral quasispecies, which can be further utilized to examine genetic interactions and to identify functional residues.

Our technique provides a sensitive and accurate tool to study the evolutionary trajectory of viral quasispecies. It permits the monitoring of a multi-drug resistance (MDR) viral sequence and epistasis within viral quasispecies - an important factor in viral evolution and adaptation [37], [38]. Highly active antiretroviral therapy (HAART) therapy is a common treatment to suppress HIV progression by utilizing a drug cocktail designed to target viral proteins at multiple essential stages of the viral life cycle. However, viral rebound can be caused by MDR HIV with extremely low occurrence frequency [9], [38]–[40]. In addition, as most drug resistant mutations compromise viral fitness, drug resistant viruses often carry additional mutations to compensate for this fitness cost [6], [10], [41]–[43]. The tag linkage approach provides an important tool to survey the genetic makeup of viral quasispecies and to estimate the risk of viral rebound and virulence by surveillance of pair-wise or even higher-order genetic interactions between mutations.

Although this study is based on HIV quasispecies samples, tag linkage approach is not limited to HIV and can potentially be applied to other viral quasispecies, such as hepatitis B virus (HBV), hepatitis C virus (HCV) and influenza virus. For example, tag linkage approach can be applied to study multi-drug resistance that are also found in naturally occurring HBV as in the case of HIV [44], [45]. This technique is also suitable for studying *cis*-elements that are prevalent in HCV due to its intrinsic replication property [46]. In addition, tag linkage approach can be utilized to examine permissive and compensatory mutations that are shown to be important in the evolution of influenza virus [47]–[49].

This technique can also be extended beyond the monitoring of viral quasispecies. One application is to examine the dynamics of CD4^+^ and CD8^+^ cells in the immune system during viral infection. They have an active role in virus detection and clearance during both acute and chronic infection. During the establishment of persistent viral infections, the immune system co-evolves with the virus [50]. A complex dynamic occurs between the heterogeneous immune populations and the evolving viral quasispecies. The medical significance of this virus-host dynamic is highlighted by a recent study describing the rise of a broadly neutralizing HIV-1 antibody from co-evolution with acute phase virus [51]. The methodology we describe here offers the research community an approach to understand the dynamic interplay between the host and virus in exquisite detail at the population level.

## Materials and Methods

### Ethics Statement

The study was approved by UCLA IRB. A chronically-infected HIV-1 patient without undergoing antiretroviral therapy was recruited from the Los Angeles area and provided written informed consent.

### Subjects and Specimen Collection

Total peripheral blood mononuclear cells (PBMCs) were isolated from the patient’s whole blood sample by standard Ficoll gradient. The plasma viral load at the time of collection was 130,234 viral copies/ml.

### Recovery of Virus from PBMCs and Virus Passaging

*Ex vivo* passaging was conducted as previously described [52]. Briefly, virus was passaged serially in primary CD4^+^ T lymphocytes from an HIV-1-uninfected donor [53]. After each passage of ∼7 days, supernatant virus was collected, titered, and used to infect fresh cells with an MOI of 1.

### DNA Library Preparation for Tag Linkage Assembly

To extract the viral genomic DNA, cell pellets of 200,000 cells were resuspended in PBS and genomic DNA was extracted using the DNeasy Tissue DNA Isolation Kit (Qiagen). DNA was recovered by PCR using the primer set: 5′-GCG GAG GCT AGA AGG AGA GAG ATG G-3′ and 5′-CAT CAC CTG CCA TCT GTT TTC CAT A-3′. The forward Illumina sequencing priming site was added to the 5′ end of the DNA sample by PCR using the primer set: 5′-AGA TCG GAA GAG CGT CGT GTA GGG GCG GAG GCT AGA AGG AGA GAG ATG-3′ and 5′- GTT TAA CTT TTG GGC CAT CCA TTC CTG GC-3′. Then, the constant region, a NotI restriction enzyme site and a 13 nucleotide tag of random ‘N’ sequence was added to the 5′ end of the DNA sample by another PCR using the primer set: 5′-ACA TAG ATA CTA TGC GGC CGC NNN NNN NNN NNN NAG ATC GGA AGA GCG TCG TGT AGG G-3′ and 5′- GTT TAA CTT TTG GGC CAT CCA TTC CTG GC-3′. The concentration of the tagged DNA sample was measured using NanoDrop 1000 spectrophotometer (Thermo Fisher Scientific). This concentration was used as a reference to calculate the dilution-fold in the subsequent complexity control step. In the complexity control step, ∼300,000 copies of tagged DNA sample were used as the input for PCR using the primer set: 5′-CAC ATA GAT ACT ATG CGG CCG C-3′ and 5′-GTT TAA CTT TTG GGC CAT CCA TTC CTG GC-3′. This complexity was calculated based on a ∼50-fold coverage for individual viral sequence with 30 Gb expected sequencing output per viral quasispecies sample. This was followed by 12 PCR using the universal forward primer, 5′-CAC ATA GAT ACT ATG CGG CCG C-3′, and the reverse primers as stated in Table S2 in File S1 to add the XhoI restriction enzyme site on the 3′ end using the product of the complexity control step as template. Consecutive PCR pools should have a different product size approximately corresponding to the sequencing read length minus 80 bp (Table S3 and S4 in File S1). From this step forward, the 12 pools were processed independently until sample combination at the high-throughput sequencing step. The products were then subjected to double digestion by NotI and XhoI. NotI and XhoI were chosen because they were not present in the consensus sequence of the target DNA template region. A small insert, which could serve as the population ID, was prepared by annealing 5′-GGC CCG ACG TAA CGA T-3′ and 5′-TCG AAT CGT TAC GTC G-3′, each with a phosphate group attached at the 5′ end. Ligation was performed using the small insert to DNA sample at the molar ratio as stated in Table S2 in File S1. One unit of T4 DNA ligase (Life Technolgies) was used in each ligation reaction. The reaction condition followed manufacturer’s instructions. All ligations were performed overnight at 20°C in 100 uL total reaction volume. The ligated products were used as the templates for PCR to add the 5′ flow cell adapters and the reverse read Illumina sequencing priming site using the universal forward primer, 5′-AAT GAT ACG GCG ACC ACC GAG ATC TAC ACT CTT TCC CTA CAC GAC GCT CTT CCG-3′, and the reverse primers as stated in Table S2 in File S1. The 3′ Illumina flow cell adapters were then added by PCR using the primer set: 5′-AAT GAT ACG GCG ACC ACC G-3′ and 5′- CAA GCA GAA GAC GGC ATA CGA GAT CGG TCT CGG CAT TCC TGC TGA ACC GCT CTT CCG-3′. The resultant amplicons from all 12 pools were then mixed. High-throughput sequencing was done by an Illumina HiSeq 2000 machine with an equivalent of 0.75 lane per sample and 2×100 bp paired-end reads. All PCRs in this study were performed using KOD DNA polymerase with 1.5 mM MgSO_4_, 0.2 mM of each dNTP (dATP, dCTP, dGTP, and dTTP) and 0.4 uM of forward and reverse primer. PCR extensions were performed with 50 seconds per kb at 68°C. Annealing temperature for a given PCR was 5°C below the lowest melting temperature of the pair of primers. All primers in this study were designed to target conserved regions within the quasispecies which were determined by clonal sequencing of the sampled viral sequences. This sequencing library preparation could potentially be adapted to study viral RNA using a reverse transcription primer tag as decribed by Jabara et al [54]. Raw sequencing data have been submitted to the NIH Short Read Archive under accesion number: SRP032753.

### Clonal Sequencing

After recovering the DNA by PCR as described above, the amplicon was inserted into target p83-2 plasmid using In-Fusion kit (Clontech). Twenty clones were randomly selected and subjected to capillary sequencing (Laragen).

### Data Analysis

Sequencing reads were mapped by BWA with 8 mismatches allowed [55]. Pair-end reads containing two or more short inserts (barcodes) were discarded. Error-correction was performed as described previously to distinguish true mutation from sequencing error [21]. The error-correction step grouped all reads sharing the same tag and mapped to the same region into a read cluster that was further conflated into a “error-free” read. As described in Kinde et al. [21], most reads sharing the same tag should share the mutation pattern during mapping. In contrast, a sequencing error would have a low occurrence frequency within a read cluster and could be distinguished from true mutations. Through this process, sequencing error would be corrected to generate an “error-free” read. Read cluster with a size of <3 reads were discarded to increase the confidence in generating an “error-free” read. Since intermolecular concatenation at the ligation was observed, a mutation that existed in 45% of the reads within a conflated read cluster that also shared the same tag was considered as a true mutation. The correlation between technical replicates indicated that intermolecular concatenation did not pose a major barrier in the accuracy of viral quasispecies assembly. Nonetheless, further application should adjust the ligation reaction volume to decrease the intermolecular concatenation during ligation (circularization step). Next, “error-free” reads that shared the same tag were assembled into a contiguous sequence, which represented a single viral sequence. Data processing and analysis were conducted by custom Python scripts. All scripts are available upon request.

### Phylogenetic Tree Construction

ClustalX was used to create the neighbor-joining phylogenetic tree [56]. The phylogenetic tree was mid point-rooted and displayed by FigTree.

### Linkage Disequilibrium

We used the r^2^ correlation to quantify linkage disequilibrium between two SNPs. R^2^ was computed as per convention. Briefly, r^2^ = (P*_AB_* − P*_A_* x P*_B_*)^2^/(P*_A_* × P*_B_* × (1 P*_A_*) × (1 P*_B_*)), where P*_AB_* represented the occurrence frequency of viral sequences that carry both SNP A and SNP B; P*_A_* represented the occurrence frequency of viral sequences that carry SNP A; P*_B_* represented the occurrence frequency of viral sequences that carry SNP B.

### DNA Library Preparation for Error-free Sequencing

Gag-pol region was PCR amplified using the primer set: 5′- GAC TAG CGG AGG CTA GAA GGA GAG AG-3′ and 5′-CAT GTT CTT CTT GGG CCT TAT CTA TTC-3′. The resultant DNA product was sheared to around 200 bp to 600 bp by sonication using the Sonic Dismembrator Model 100 (Fisher Scientific). Dismembrator was set to power level four and samples were pulsed three times for 10 seconds. Samples were kept on ice for 45 seconds in between pulses. End repair and 3′ dA-tailing were performed respectively by end repair module and dA-tailing module (New England BioLabs). The DNA product was then ligated to an Y-shape adaptor carrying a nine-nucleotide tag of random ‘N’ sequence. As a result, each ligated product contained an 18-nucleotide tag, nine from each of the 5′ and 3′ end. Y-shape adaptor was prepared by annealing two oligonucleotides: 5′-CGC GTA TCC ATG GCA NNN NNN NNN GCC AGA TCG GAA GAG CGG TTC AGC AGG AAT GCC GAG-3′ and 5′-ACA CTC TTT CCC TAC ACG ACG CTC TTC CGA TCT GGC-3′. Then, the annealed product was treated with Klenow Fragment (New England BioLabs) and digested with BciVI. An estimated copy of around 10 millions of ligated products were amplified by primer set: 5′-AAT GAT ACG GCG ACC ACC GAG ATC TAC ACT CTT TCC CTA CAC GAC GCT CTT CCG-3′ and 5′-CAA GCA GAA GAC GGC ATA CGA GAT CGG TCT CGG CAT TCC TGC TGA ACC GCT CTT CCG-3′. The resultant DNA product was submitted for 2×100 bp paired-end sequencing on one lane of Illumina HiSeq 2500 machine.

### Quasispecies Reconstruction by ShoRAH and QuasiRecomb

“Error-free” reads were generated as described above. Here, a mutation that existed in 95% of the reads within a conflated read cluster that also shared the same tag was considered as a true mutation. Reads were mapped by BWA with 8 mismatches allowed [55]. All reads were treated as single end read. “Error-free” mapped reads were processed by ShoRAH version 0.6 with a window size of 40, a window shift of 1 and default settings for other parameters [13]. Quasispecies reconstruction by QuasiRecomb was performed by default setting [12]. Due to the huge memory requirement of QuasiRecomb, 500,000 mapped reads were randomly sampled and processed. Further increase the number of input reads generated memory error. To limit the false positive rate, a refinement reconstruction was performed using ‘-refine’ option. We employed ‘-conservative’ option for high confidence haplotype reconstruction to identify major haplotypes.

## Supporting Information

File S1 **Figures S1 and S2 and Tables S1–S4.** Figure S1. Concept of complexity control. In this graphical demonstration, we employ a simple example with five amplicons and 30 reads sequenced. A total of nine viral sequences are present in the viral quasispecies with the genotype being A or B. The colored boxes represent the tag for distinguishing an individual viral sequence within the viral quasispecies. Different colors represent different nucleotide sequences in individual tags. The white boxes represent individual viral sequences. During the Amplicon generation and sequencing step, each column of amplicons represents one genomic region of the viral quasispecies. (A) Complexity is too high (complexity = 9) where each viral sequence is not sufficiently covered. (B) Complexity is too low (complexity = 1) where each viral sequence is excessively covered and therefore, there is a waste of sequencing capacity. (C) Complexity is well-controlled (complexity = 3) such that individual viral sequences are sufficiently covered for sequencing error correction and for sequence assembly. Figure S2. Key step in the experimental design. (A) A detailed representation that shows the cassette sequence in Figure 1B. (B) A detailed representation that shows the cassette sequence after ligation.
(PDF)
