# Supplementary material for: HIV-1 Quasispecies Delineation by Tag Linkage Deep Sequencing
Source: PLoS One. 2014 May 19;9(5):e97505. doi: 10.1371/journal.pone.0097505 (PMC4026136; doi:10.1371/journal.pone.0097505)
Supplement: File S1 — Figures S1 and S2 and Tables S1–S4. Figure S1. Concept of complexity control. In this graphical demonstration, we employ a simple example with five amplicons and 30 reads sequenced. A total of nine viral sequences are present in the viral quasispecies with the genotype being A or B. The colored boxes represent the tag for distinguishing an individual viral sequence within the viral quasispecies. Different colors represent different nucleotide sequences in individual tags. The white boxes represent individual viral sequences. During the Amplicon generation and sequencing step, each column of amplicons represents one genomic region of the viral quasispecies. (A) Complexity is too high (complexity = 9) where each viral sequence is not sufficiently covered. (B) Complexity is too low (complexity = 1) where each viral sequence is excessively covered and therefore, there is a waste of sequencing capacity. (C) Complexity is well-controlled (complexity = 3) such that individual viral sequences are sufficiently covered for sequencing error correction and for sequence assembly. Figure S2. Key step in the experimental design. (A) A detailed representation that shows the cassette sequence in Figure 1B. (B) A detailed representation that shows the cassette sequence after ligation. (PDF) [file pone.0097505.s001.pdf]

Supporting information

HIV-1 Quasispecies Delineation by Tag Linkage Deep Sequencing

Nicholas C. Wu[1,2], Justin De La Cruz[3], Laith Q. Al-Mawsawi[1], C. Anders Olson[1], Hangfei Qi[1], Harding H. Luan[1], Nguyen Nguyen[1], Yushen Du[1], Shuai Le[4], Ting-Ting Wu[1], Xinmin Li[5], Martha J. Lewis[6,7], Otto O. Yang[3,6,7,8], Ren Sun[1,2,7,*]

[1]Department of Molecular and Medical Pharmacology, David Geffen School of Medicine, University of California, Los Angeles, CA 90095, USA

[2]Molecular Biology Institute, University of California, Los Angeles, CA 90095, USA

[3]Department of Microbiology, Immunology, and Molecular Genetics, David Geffen School of Medicine, University of California, Los Angeles, CA 90095, USA

[4]Department of Microbiology, Third Military Medical University, Chongqing, 400038, China

[5]Department of Pathology and Laboratory Medicine, David Geffen School of Medicine, University of California, Los Angeles, CA 90095, USA

[6]Division of Infectious Diseases, Department of Medicine, David Geffen School of Medicine, University of California, Los Angeles, CA 90095, USA

[7]AIDS Institute, University of California, Los Angeles, CA 90095, USA

[8]AIDS Healthcare Foundation, Los Angeles, CA 90028, USA

[*]To whom correspondence may be addressed. Phone Number: 1-310-794-5124. Email: RSun@mednet.ucla.edu

This file includes: Figure S1-2 and Table S1-4

Wu et al. Supplemental Fig. 1

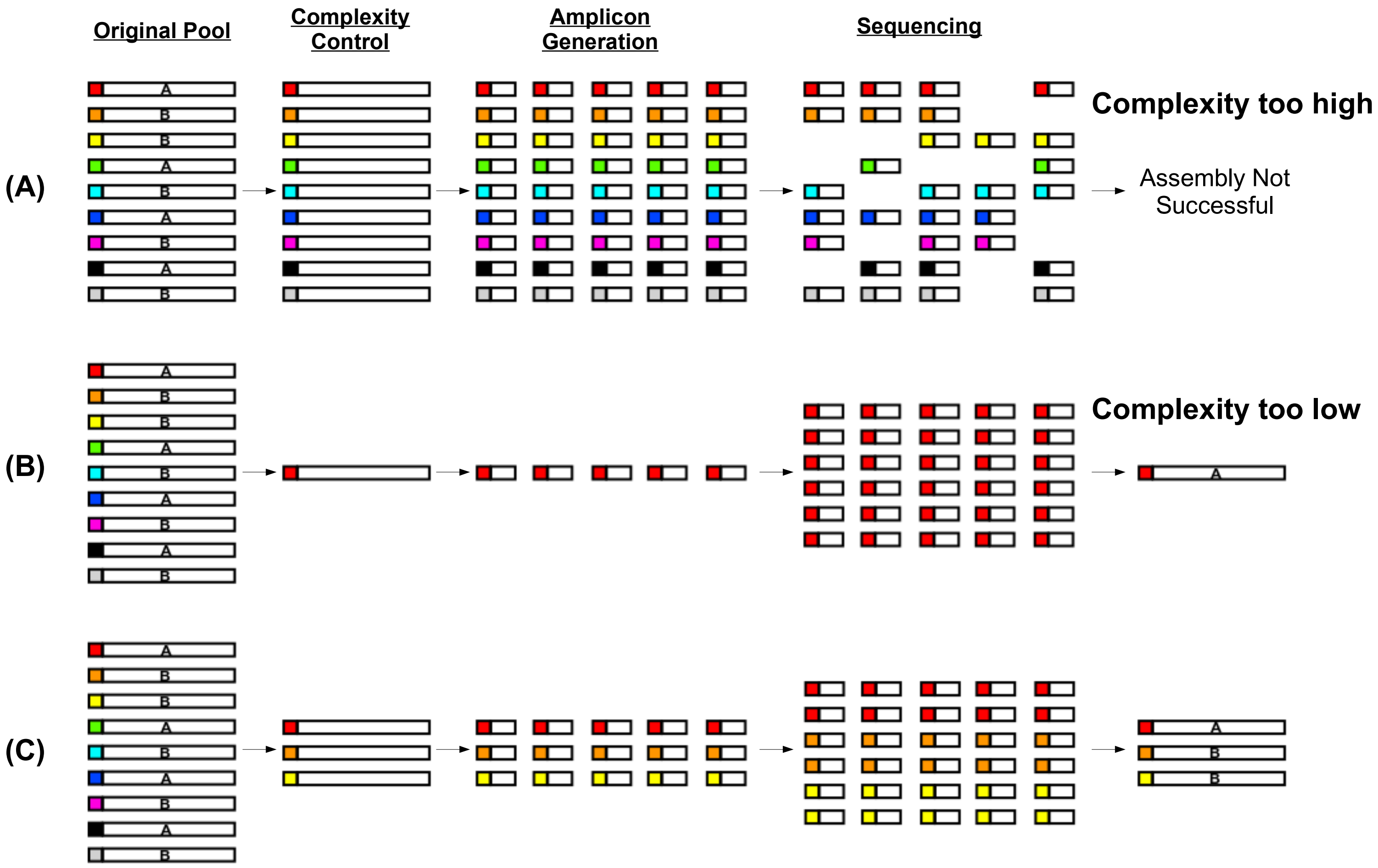

Wu et al. Supplemental Fig. 2

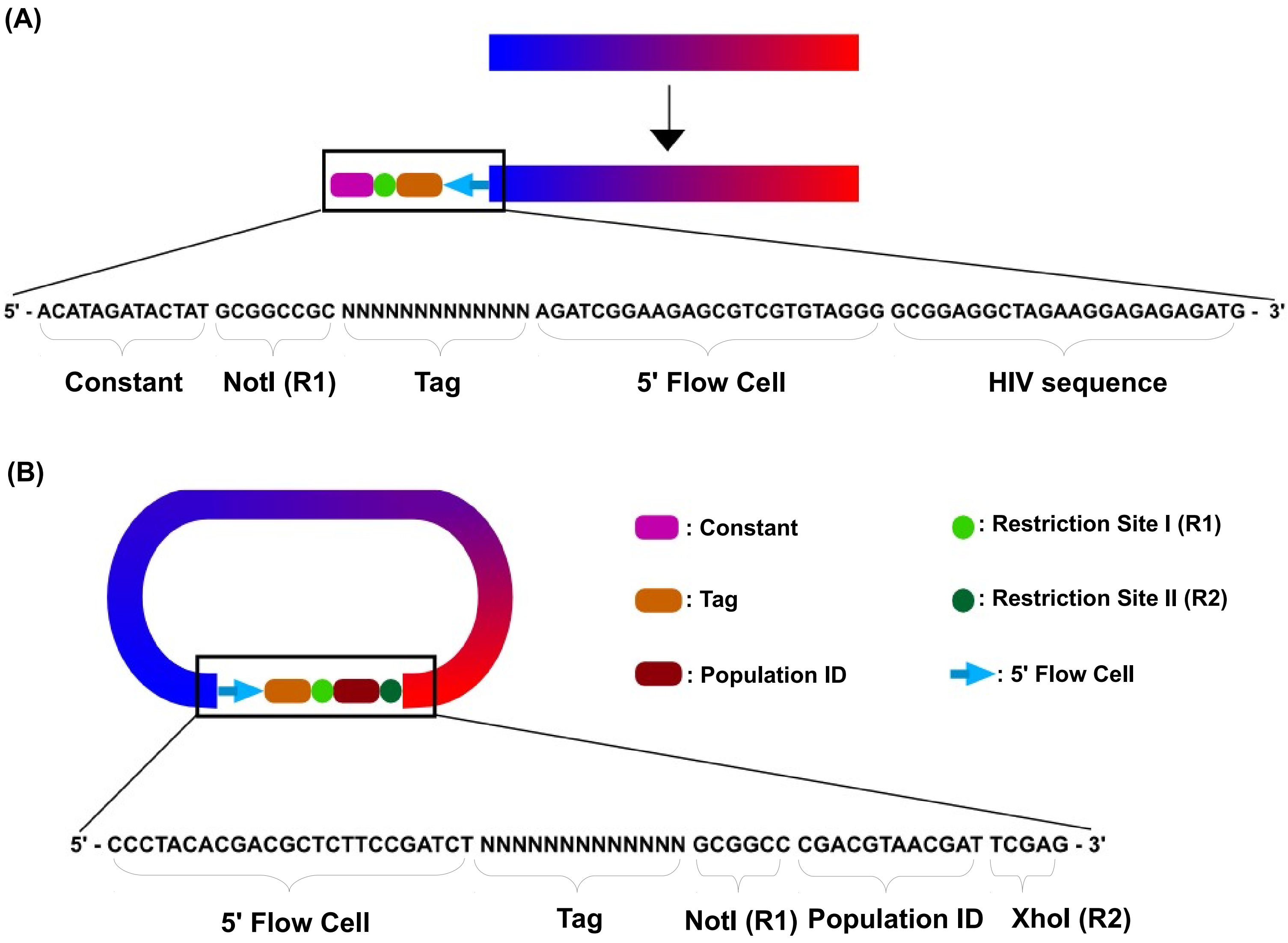

Table S1. Data Summary

	in vivo	*ex vivo*
Assembled Sequence	54583	228936
Sequence with Occurrence > 2	47083	223966
Unique DNA Sequence[a]	2672	1983
Number of Variable Position[a]	75	255
Number of Unique Gag Protein Sequence[a b]	42	201
Number of Unique Gag DNA Sequence[a b]	1381	851

[a]Based on the analysis on sequences with occurrence > 2.

[b]Only the region from aa. 139 to 507 was being analyzed.

Table S2. Primer sequences and ligation ratios.

	Reverse Primer for Pre-ligation PCR	Ligation Ratio[a]	Reverse Primer for Post-ligation PCR
Pool 1	ATTCGTCTCGAGAATCCTGTG GGGTGGCTC	50:1	TGCTGAACCGCTCTTCCGATCT CCCTATAGTGCAGAACATGC
Pool 2	ATTCGTCTCGAGCTATCCCATT CTGCAGCTT	50:1	TGCTGAACCGCTCTTCCGATCT TTCAGCCCAGAAGTGATACC
Pool 3	ATTCGTCTCGAGTCATCCATCC TATTTGTTCC	50:1	TGCTGAACCGCTCTTCCGATCT TAAAAGAGACCATCAATGAGG
Pool 4	ATTCGTCTCGAGCTTGTCTTAT GTCCAGAATG	50:1	TGCTGAACCGCTCTTCCGATCT GGAACTACTAGTACCCTTC
Pool 5	ATTCGTCTCGAGCGCATTCTG GACCAACAAGG	50:1	TGCTGAACCGCTCTTCCGATCT GGATGTATAGCCCTGTCAG
Pool 6	ATTCGTCTCGAGAACTCTTGCT TTATGGCCGG	50:1	TGCTGAACCGCTCTTCCGATCT GTAAAAAATTGGATGACAGAAA
Pool 7	ATTCGT CTCGAG CACTTAACAATCTTTCTTTGGT	100:1	TGCTGAACCGCTCTTCCGATCT AYTAGAAGAAATGATGACAGC
Pool 8	ATTCGT CTCGAG TCAGWGCACTCTTTCATTTG	100:1	TGCTGAACCGCTCTTCCGATCT GCAGAAAGGCAATTTTAGGA
Pool 9	ATTCGT CTCGAG AACCTGAAGCTCTCCTCTG	200:1	TGCTGAACCGCTCTTCCGATCT ATGTGGAAAGGAAGGACAC
Pool 10	ATTCGT CTCGAG CGTTGCCAAAGAGTGATCT	200:1	TGCTGAACCGCTCTTCCGATCT CAGAACCATCAGCCCCAC
Pool 11	ATTCGT CTCGAG TTTTTGGTYTCCATCTYCCTG	200:1	TGCTGAACCGCTCTTCCGATCT CCTCCYTTAGCTTCCCTC
Pool 12	ATTCGT CTCGAG CAGGTGTAGGTCCTACTAAT	200:1	TGCTGAACCGCTCTTCCGATCT TATTAGAAGATATGGAGTTGC

[a]Ligation ratio represents the molar ratio of small insert and the corresponding DNA sample.

[b]The primer shown in the table is for the sample from *in vivo*, the primer, TGCTGAACCGCTCTTCCGATCTGCAGAAAGGCAAGTTTAGGA , is used for the sample from *ex vivo*.

Table S3. Information for primer design.

Total Paired-End Read Length (bp)	200
Restriction Site I (bp)	6 to 8
Restriction Site II (bp)	6 to 8
Barcode (bp)	13
Population ID (bp)	8
PCR 5' Priming Region (bp)	~20
PCR 3' Priming Region (bp)	~20
Informative Region	200 – (6 to 8) x 2 – 13 – 8 – 20 – 20 = ~120

Table S4. Amplicon information.

	Start[a]	End[b]	5' Priming Length[c]	3' Priming Length[d]
Amplicon1	417	571	18	20
Amplicon2	511	662	19	20
Amplicon3	623	772	20	21
Amplicon4	733	883	20	19
Amplicon5	845	999	20	19
Amplicon6	958	1107	20	22
Amplicon7	1047	1196	22	21
Amplicon8	1155	1304	20	20
Amplicon9	1266	1415	19	19
Amplicon10	1379	1528	19	18
Amplicon11	1492	1641	21	18
Amplicon12	1600	1749	20	21

[a]Start: The first base of the amplicon including priming region

[b]End: The last base of the amplicon including priming region

[c]5' Priming Length: The length in the 5' end of the amplicon that is attributed by the primer

[d]3' Priming Length: The length in the 3' end of the amplicon that is attributed by the primer
